# A novel collagen hydrogel loaded with primary brown sugar for accelerating burn wound healing by a synergistic mechanism

**DOI:** 10.1093/rb/rbag113

**Published:** 2026-06-03

**Authors:** Ziying Guan, Liren Wei, Liaoyuan Han, Jian Lu, Yanfei Tan, Qingrong Wei

**Affiliations:** National Engineering Research Center for Biomaterials (NERCB), Sichuan University, Chengdu 610065, China; College of Biomedical Engineering, Sichuan University, Chengdu 610065, China; National Engineering Research Center for Biomaterials (NERCB), Sichuan University, Chengdu 610065, China; College of Biomedical Engineering, Sichuan University, Chengdu 610065, China; National Engineering Research Center for Biomaterials (NERCB), Sichuan University, Chengdu 610065, China; College of Biomedical Engineering, Sichuan University, Chengdu 610065, China; National Engineering Research Center for Biomaterials (NERCB), Sichuan University, Chengdu 610065, China; College of Biomedical Engineering, Sichuan University, Chengdu 610065, China; National Engineering Research Center for Biomaterials (NERCB), Sichuan University, Chengdu 610065, China; College of Biomedical Engineering, Sichuan University, Chengdu 610065, China; National Engineering Research Center for Biomaterials (NERCB), Sichuan University, Chengdu 610065, China; College of Biomedical Engineering, Sichuan University, Chengdu 610065, China

**Keywords:** brown sugar, collagen, burn wound, regenerative healing, synergistic effect

## Abstract

Severe skin burns present complex clinical challenges, including delayed healing and scar formation. As the first-extracted product of sugarcane, brown sugar has a long application history in Chinese traditional medicine. Inspired by its medicinal benefits, this study developed a collagen-based hydrogel co-loaded with brown sugar and epicatechin (COL-BS-EC) and evaluated its synergistic enhancement of therapeutic potential for promoting burn regenerative healing by comparison with comprehensive control groups and explored its relevant mechanisms involving the synergistic actions of non-sucrose components. Such a designed hydrogel showed potent species-selective antibacterial activity, particularly against *S. aureus*, and high DPPH radical scavenging. *In vitro* assays confirmed significant reductions in pro-inflammatory TNF-α and increases in anti-inflammatory IL-10, alongside enhanced fibroblast migration and proliferation. The experimental results from burn models on mice and rats revealed that COL-BS-EC group accelerated the wound closure by reducing the residual wound area to about 6% on Day 14, promoted organized collagen deposition of 32% versus 13% in the control group and doubled neo vessel density. This study provides a modern scientific validation for the synergistic therapy efficacy of brown sugar for wound tissue and explores the potential of this concept applied to biomaterial design for clinical burn wounds.

## Graphical abstract

**Graphical Abstract:**
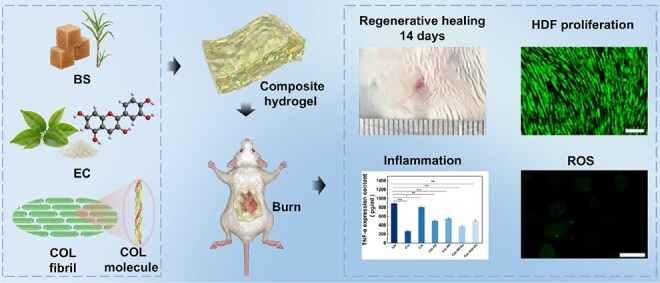

## Introduction

Severe skin burns are one of the significant wound care problems in clinics, leading to a heightened susceptibility to infections, substantial functional impairment and scar formation, which impose a substantial burden on individuals and communities [1]. The pathophysiological conditions of burn injuries, particularly those classified as deep second-degree or more severe, are highly intricate and extend well beyond mere disruption of the skin’s protective barrier [2, 3]. The healing processes of these burns are generally confronted with multiple interrelated challenges, such as a persistent and unregulated inflammatory reaction [4], intense oxidative stress, a high risk of bacterial invasion [5] and even the consequent malfunctions of critical regenerative cells, leading to the delay of tissue repair and angiogenesis [6]. Therefore, an effective therapeutic approach is needed to address these difficult situations collectively to achieve regenerative wound healing rather than mere wound closure [6].

The limitations of existing dressings such as gauze and petroleum jelly, along with the high cost and single-function bottlenecks of some advanced dressings, have driven researchers to explore bioactive hydrogels as interactive platforms for wound management [7, 8]. Collagen-based hydrogels are notable for their innate biocompatibility, degradability, facilitating cell adhesion and proliferation and utilized as the extracellular matrix [9, 10]. Their porous three-dimensional structures efficiently absorb exudate, sustain a moist environment and permit substance exchange, rendering these hydrogels as excellent delivery vehicles for therapeutic agents [11, 12]. Loading collagen (COL) frameworks with bioactive compounds to establish a versatile system is a significant approach in burn therapy [13]. Especially an integration of natural compounds with defined functions into wound dressing matrices provides a highly promising treatment [14, 15]. Among them, plant-derived polyphenolic compounds have garnered significant interest in the field of wound healing [16]. Epicatechin (EC), a flavan-3-ol abundantly present in green tea and cocoa, has been established as a potent antioxidant and anti-inflammatory agent [17]. This study incorporates epicatechin as a highly effective antioxidant component within our designed dressing system, aim to mitigate the exacerbated oxidative stress characteristic of burn wounds.

However, the complex process of wound healing, particularly regenerative rather than scar-forming healing in a shorter period, necessitates an efficacious and synergistic therapeutic strategy [18]. The limitation of single-functional dressing has led to focus on natural products with a long history of being used as both medicine and food, especially brown sugar (BS), which comprises diverse functional components [19, 20]. As the first-extracted product of sugarcane, the outstanding efficacy of BS for wound therapy has been recorded in the classical medical texts of ancient China [21]; yet its modern scientific validation and underlying mechanisms remain unexplored largely, with its efficacy often being simply ascribed to hyperosmolarity or a single component of sucrose self [22]. BS comprises not only sucrose but also a natural complex abundant in minerals, vitamins, organic acids and diverse phenolic compounds [19, 23]. We hypothesize that the significant comprehensive therapeutic efficacy of brown sugar documented in traditional Chinese medical literature [24] should originate from the synergistic enhancement produced by the synergistic actions of the diverse components within BS.

Based on the above hypothesis, the present study integrated the respective advantages of BS and bioactive collagen to design a collagen-based hydrogel as an advanced dressing material loaded with BS and EC. By setting up comprehensive control groups, this work has tried to verify and reveal the remarkable behaviors and underlying mechanisms of BS in promoting a regenerative repair of burn wounds, validating the above hypothesis experimentally. From this study, we aim at achieving a combination of deeply exploring the treasure of traditional Chinese medicine and the functional design of modern biomedical materials.

## Materials and methods

### Materials

All the medical-grade collagen (type I) used in this study was produced from calfskin by our laboratory. The brown sugar (BS) applied in this study is natural and additive-free original flavor brown sugar, which was produced by Guangxi Lingmeng Liuxiang Sugar Industry (Guigang City, Guangxi Zhuang Autonomous Region). Epicatechin (EC) was purchased from Macklin. Dulbecco’s Modified Eagle Medium (DMEM, Gibco), fetal bovine serum (FBS, Gibco), trypsin (Gibco), penicillin-streptomycin solution (Gibco) and phosphate-buffered saline (PBS, Gibco) were employed in cell-culture experiments. Isoflurane anesthesia was used for animal experiments. Chemicals such as phosphoric acid were used directly as analytical-grade reagents. Ultrapure water utilized in all experimental procedures was produced via Milli-Q system.

### Preparation of functional collagen hydrogel

Highly purified soluble collagen was dissolved in phosphoric acid (pH 2.5) to obtain an acidic collagen solution at a concentration of 7 mg/mL. Under moderate stirring, 500 μL of disodium hydrogen phosphate solution (0.5 mol/L) was added dropwise to the collagen solution, and then the pH of this mixture was gradually adjusted to 7.5 with sodium hydroxide solution (2.0 mol/L). Next, EC solution (1.0 mol/L) was added dropwise to the collagen solution, and stirred for 5 min, followed by the dropwise addition of brown sugar solution (1.0 mol/L) or sucrose (SUC) solution (1.0 mol/L). Stirring was continued for 5 min, then the mixture was injected into molds and incubated at 37°C for 12 h to fabricate hydrogel samples. All experimental procedures involving collagen were performed at 4°C.

In the preliminary experiments, concentration gradients were separately established for COL, BS and EC. The COL gradient ranged from 1.0–6.0 mg/mL (1.0, 2.0, 3.0, 4.0, 5.0 and 6.0 mg/mL), the BS gradient from 0.6–6.0 mg/mL (0.6, 1.2, 2.4, 4.8 and 6.0 mg/mL) and the EC gradient from 0.1 to 0.8 mg/mL (0.1, 0.2, 0.4, 0.6 and 0.8 mg/mL). The mechanical properties of hydrogels with different formulations were evaluated by dynamic mechanical analysis (DMA). Concurrently, antioxidant activity at 24 h, 48 h and 72 h was determined by DPPH free radical scavenging assays, and cell viability at 24 h and 48 h was evaluated by CCK-8 proliferation assays using L929 cells. These data were integrated to identify the optimal concentrations for each component in the composite hydrogel.

Based on the analysis of preliminary results, the final concentrations were determined as follows: COL at 6.0 mg/mL, BS at 4.8 mg/mL, EC at 0.2 mg/mL and pure sucrose at 4.0 mg/mL. Ensure that the final concentration of collagen in the hydrogel is 6.0 mg/mL, the final concentration of epicatechin is 0.2 mg/mL and the final concentration of brown sugar is 4.8 mg/mL. The hydrogel samples were designated as COL, COL-EC, COL-BS, COL-BS-EC and COL-SUC-EC, respectively, according to the components in the hydrogel system.

### Scanning electronic microscopy

The microstructure of the hydrogels was characterized using a field-emission scanning electron microscope (SEM, S-4800, Hitachi, Japan). The hydrogel samples were freeze-dried and then attached to conductive adhesive tape and sputter-coated with gold for SEM observation.

### Mechanical characterization

The viscoelastic properties, including storage modulus (*G*′), loss modulus (*G*″) and Tan delta (*G*″/*G*′) were measured on a dynamic mechanical analyzer (DMA, TA Instruments Q800, USA) at room temperature. Cylindrical hydrogel samples (9 mm in diameter and 5 mm in height) were subjected to compression testing with a preload force of 0.02  N and an amplitude of 40 μm at dual frequencies of 1 and 2 Hz. Each measurement was repeated in triplicate at each frequency. Accurate sample dimensions were measured using a digital vernier caliper for the diameter (two perpendicular measurements) and using the axial compression system for the height as defined by the preload force.

### Swelling test of hydrogel

The swelling performance of hydrogels was evaluated by calculating the weight change. First, pre‑weighed hydrogel samples (*W*_0_) were immersed in PBS (pH 7.4) at room temperature. At predetermined time intervals, the hydrogels were removed and the excess water on the surface gently blotted with filter paper. The weight of the swollen hydrogels (*W*_s_) was then measured. The swelling ratio was calculated using the following equation:


$$\mathrm{Swelling}\;\;\mathrm{ratio}\;\left(\%\right)=\left[\left(W_{s}-\;W_{0}\right)/\;W_{0}\right]\times 100\mathrm{\%.}$$

### Antibacterial activity *in vitro*

The antibacterial properties of the hydrogels were evaluated against two model bacteria, Staphylococcus aureus (*S. aureus*) and Escherichia coli (*E. coli*), using the colony counting method. First, 100 µL of bacterial suspension (10^8^ CFU/mL) was uniformly spread onto the hydrogel surface. After 12 h of co-culture with the hydrogels, the bacterial suspensions were collected and serially diluted to 10^1—^10^2^ CFU/mL. Subsequently, 5 µL of each diluted sample was spotted onto Luria-Bertani (LB) agar plates, and bacterial viability was quantitatively analyzed by enumerating the CFU. All plates were then incubated at 37°C for 12 h to allow colony formation. Finally, the antibacterial effect was evaluated by calculating the bacterial inhibition rate.

### Free radical scavenging activity

To evaluate the 2,2-diphenyl-1-picrylhydrazyl (DPPH) radical scavenging activity, 500 μL of hydrogel suspension or 500 μL of ultrapure water (control) was mixed with 0.1 mM DPPH solution prepared in anhydrous ethanol, and the mixture was incubated at room temperature in the dark. The absorbance (Abs) at 517 nm was measured using a UV-visible spectrophotometer. The DPPH radical scavenging rate was calculated using the following equation:


$$\mathrm{Scavenging}\;\mathrm{rate}\;\left(\%\right)\;=\;\left[\left(Abs_{\mathrm{con}}-\;Abs_{\mathrm{Hydrogel}}\right)/\;Abs_{\mathrm{con}}\right]\times 100\mathrm{\%.}$$

### ROS-scavenging *in vitro*

The intracellular reactive oxygen species (ROS) scavenging ability was evaluated in RAW 264.7 cells. Cells were seeded in 8-well chamber slides at a density of 5 × 10^3^ cells/well and preincubated for 24 h at 37°C. Subsequently, the culture medium was replaced with various hydrogel extracts prepared in medium containing lipopolysaccharide (LPS, 3 μg/mL), followed by a further 12 h incubation under standard conditions to induce an inflammatory response. After gentle washing with PBS, cells were sequentially stained with Hoechst and 2',7'-dichlorodihydrofluorescein diacetate (DCFH-DA) fluorescent probes. First, cells were incubated in Hoechst-containing medium for 10 min in the dark for nuclear staining. Following another wash, cells were incubated in DCFH-DA-containing medium for 30 min in the dark to detect intracellular ROS levels. After staining, fluorescence images were immediately acquired using confocal laser scanning microscopy (CLSM), and semi-quantitative analysis of fluorescence intensity was conducted using ImageJ software. The following control groups were established: normal macrophages incubated with DMEM only and LPS-activated macrophages without hydrogel extract treatment.

### Anti-inflammatory effect *in vitro*

To evaluate the anti-inflammatory activity of the hydrogels, RAW 264.7 cells were seeded in 96-well plates at a density of 1 × 10^4^ cells/well and preincubated for 24 h in an incubator at 37°C with 5% CO_2_. After pretreatment, the medium was replaced with different hydrogel extracts containing LPS (5 μg/mL) and the cells were incubated for 24 h. The cell culture supernatants were then collected, and the expression levels of the pro-inflammatory cytokine TNF-α and the anti-inflammatory cytokine IL-10 were quantitatively analyzed using enzyme-linked immunosorbent assay (ELISA) kits.

### Cell migration

This study used mouse epidermal fibroblasts (L929) to perform scratch wound healing assays. Initially, L929 cells were seeded in 6-well plates at a density of 5 × 10^4^ cells/well and incubated for 24 h. Subsequently, a uniform straight scratch was made in the cell monolayer of each well. Thereafter, the cells were treated with 2 mL of different hydrogel extracts and incubated for 24 or 48 h to evaluate migratory capacity.

### Cell proliferation and live/dead staining

This study used mouse epidermal fibroblasts (L929) and human dermal fibroblasts (HDF) to evaluate the biocompatibility of hydrogels. Hydrogels from different groups were uniformly coated onto the bottom of 24-well plates, followed by seeding L929 and HDF cells onto the surfaces of the hydrogels at a density of 1 × 10^5^ cells/well. The hydrogels with seeded cells were incubated in a 37°C, 5% CO_2_ culture incubator for 3 and 5 days, respectively. Fluorescein diacetate (FDA) and propidium iodide (PI) were used to stain viable and dead cells, respectively. Cell morphology was observed, and images were captured using a confocal laser scanning microscope (CLSM), while cell viability was quantitatively determined using the cell counting kit-8 (CCK-8) assay kits. Cells without any treatment served as the positive control, while blank wells containing only medium acted as the negative control. The relative cell survival rate was calculated using the following equation:


$$\mathrm{Cell}\;\mathrm{survival}\;\mathrm{rate}\;\left(\%\right)\;=\;\left[\left(Abs_{\mathrm{sample}}-\;Abs_{\mathrm{negative}}\right)/\left(Abs_{\mathrm{positive}}-\;Abs_{\mathrm{negative}}\right)\right]\times 100\mathrm{\%.}$$

### *In vivo* wound healing

All animal experiments were conducted in accordance with the guidelines and regulations approved by the medical ethics committee of Sichuan University (approval number: KS20260132). All animals were housed in standard specific pathogen-free animal facilities.

This study successfully established a deep second-degree burn model in mice. Male Kunming mice (18–22 g) were purchased from Chengdu Dossy Experimental Animal Co., Ltd. Deep partial-thickness burn wounds were created by applying a circular aluminum rod (1 cm in diameter) preheated to 100°C vertically onto the depilated dorsal skin of isoflurane-anesthetized mice for 10 s [25]. After modeling, the mice were randomly divided into four groups: blank control group (treated with normal saline), COL-BS group, COL-BS-EC group and COL-SUC-EC group. The wounds were then covered with appropriately sized hydrogel dressings, which were secured with Tegaderm transparent film (3M, USA) and medical bandages. The dressings were changed every 2 days, and wound images were captured on Days 3, 7, 10 and 14 to document the wound healing process. The wound area was quantitatively analyzed using ImageJ software. Furthermore, wound tissue samples were collected on Days 7 and 14 for subsequent histopathological examination.

To validate the generalizability of the experimental findings and minimize interspecies variability, a deep second-degree burn model was further established in Sprague-Dawley (SD) rats. Male SD rats (180–200 g) were purchased from Chengdu Dashuo Experimental Animal Co., Ltd. Two symmetrical burn wounds were created on each side of the vertebral column by applying a circular aluminum rod (1 cm in diameter) preheated to 100°C vertically onto the depilated dorsal skin for 20 s. The necrotic tissue in the burnt areas was debrided carefully after 24 h of postmodeling. The wounds were then treated with the corresponding hydrogel dressings, following the same bandaging protocol, dressing change frequency and data collection timepoints as those in the mouse model.

Wound tissue samples from each group were harvested on Days 7 and 14, respectively, followed by fixation, dehydration, embedding and sectioning. Sections from Day 7 were subjected to hematoxylin-eosin (H&E) staining and Masson staining to observe the extent of wound healing. Sections from Day 14 were subjected to H&E staining and Masson staining to examine the wound healing status and collagen deposition and CD31 immunofluorescence staining to evaluate angiogenesis. Statistical analysis of fluorescence intensity was performed using ImageJ software. Three random fields of equal area on the wound surface were selected, and the proportion of the fluorescent area was calculated.

### Statistical analyses

Data in this paper were expressed as mean ± SD deviation of the three replicates ($\bar{x}$ ± s), and one-way analysis of variance was used for comparison between groups. The significance level was set as *P *< 0.05 (*), *P *< 0.01 (**), *P *< 0.001 (***) and *n.s.* represents no significant difference.

## Results and discussion

### Synthesis and characterization of COL-BS-EC hydrogel

We identified the optimal concentrations of COL, BS and EC based on the DMA, DPPH free radical scavenging and CCK-8 assay results from the preliminary experiments. Specifically, the final concentration of collagen in the hydrogel was determined to be 6.0 mg/mL, the final concentration of BS was determined to be 4.8 mg/mL and the final concentration of epicatechin was determined to be 0.2 mg/mL. We successfully fabricated five hydrogel systems, including collagen (COL), collagen-epicatechin (COL-EC), collagen-brown sugar (COL-BS), collagen-brown sugar-epicatechin (COL-BS-EC) and collagen-sucrose-epicatechin (COL-SUC-EC).

**Figure 1:**
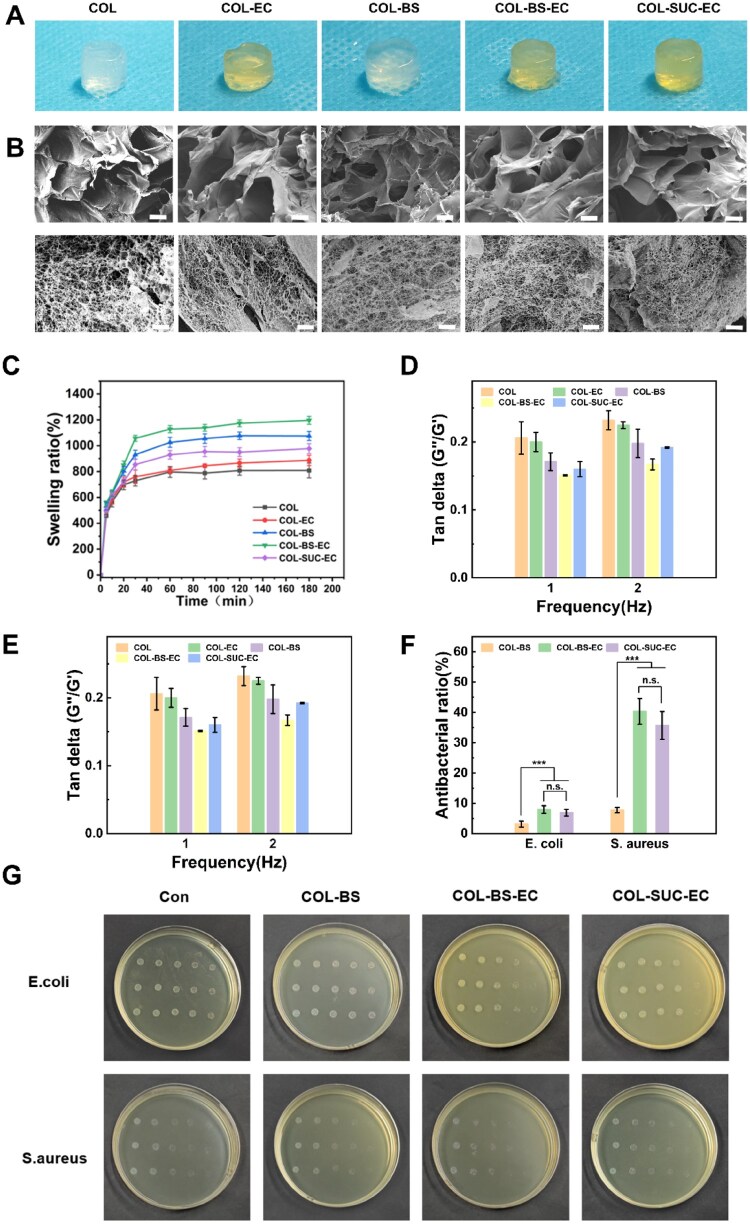
(**A**) Hydrogels appearance. (**B**) SEM images (bar = 100 μm, 10 μm). (**C**) Swelling ratios of hydrogels. (**D**) The storage modulus (*G*′), (**E**) Tan delta (*G*″/*G*′) of hydrogels of 1 and 2 Hz. (**G**) Plate images and (**F**) antibacterial ratio of *E. coli* and *S. aureus*. *P < 0.05, **P < 0.01, ***P < 0.001, *n.s*.: no significance.

As shown in Figure 1A, all groups formed cylindrical structures with excellent stability. SEM images (Figure 1B) demonstrated that all samples had an interconnected porous three-dimensional network structure. The color difference between the COL-BS and COL hydrogels confirmed the successful incorporation of BS. The EC on the hydrogel surface was oxidized upon exposure to air, producing yellow theaflavin. The hydrogels in the COL-EC, COL-BS-EC and COL-SUC-EC groups exhibited a yellow appearance, indicating the successful loading of EC. Meanwhile, the results of the major components in brown sugar and hydrogel extracts were presented in Tables 1 and 2. The results showed that sucrose was the main component in both the BS solution and the COL-BS-EC hydrogel extract. By comparing the components in these two tables, it was found that both EC and the major components in BS were detected in the hydrogel extract. These results not only confirmed that BS, together with EC, had been successfully incorporated into this collagen-based hydrogel but also directly demonstrated that such a hydrogel was composed of other multicomponents in addition to a single sucrose and EC component.

**Table 1:** Concentrations and proportions of main components in brown sugar (BS) solution.

	BS	
	Concentration (mg/mL)	Percentage (%)
Sucrose	46.25	82.59
Glucose	3.65	6.52
Fructose	3.30	5.89
Aspartic acid	1.40	2.50
Glutamic acid	0.29	0.52
Protocatechuic acid	0.85	1.52
Epicatechin	N.D.	N.D.
K	14.80 × 10^−3^	2.64 × 10^−2^
Ca	41.50 × 10^−3^	7.41 × 10^−2^
Mg	26.80 × 10^−3^	4.79 × 10^−2^

**Table 2:** Concentrations and proportions of main components in COL-BS-EC hydrogel extract.

	COL-BS-EC	
	Concentration (mg/mL)	Percentage (%)
Sucrose	3.82	79.58
Glucose	0.27	5.62
Fructose	0.24	5.00
Aspartic acid	0.11	2.31
Glutamic acid	0.024	0.44
Protocatechuic acid	0.065	1.43
Epicatechin	0.13	2.71
K	8.64 × 10^−2^	1.80 × 10^−2^
Ca	0.11	2.31 × 10^−2^
Mg	3.96 × 10^−2^	8.25 × 10^−3^

SEM images indicated no significant differences in pore size distribution among the groups, demonstrating that the incorporation of active components (EC, BS and SUC) did not damage the inherent structural integrity of the collagen matrix. This porous structure promoted cell infiltration, nutrient exchange and exudate absorption [26].

The swelling behavior of hydrogel scaffolds is important to promote tissue regeneration [26]. The hydrogel with superior water absorption capacity can rapidly absorb wound exudate, thereby creating a suitable moist environment for tissue repair [27]. Wound dressings are typically applied within the dynamic *in vivo* physiological environment. Consequently, the evaluation of their dynamic mechanical properties becomes critically important [28]. This study employed DMA at frequencies of 1 Hz and 2 Hz to simulate normal physiological pulse frequencies.

The experimental results revealed that the COL-BS-EC group exhibited the most superior performance (Figure 1C), exhibiting a swelling ratio of 1056% within 30 min, the highest storage modulus (*G*' = 24.40 ± 0.829 kPa) and the lowest Tan delta (Tan delta = 0.15 ± 0.01). These findings indicated that BS and EC achieved synergistic optimization of swelling capacity and mechanical properties through the ordered cross-linking of the collagen network. Analysis of the mechanical properties (Figures 1D and E) showed that the collagen group (COL) had a storage modulus of 6.56 ± 0.98 kPa, reflecting its loosely cross-linked collagen network. Although the COL-EC and COL-BS groups showed a better storage modulus (Figure 1D), the limited cross-linking efficiency of individual components affected the formation of a high-strength three-dimensional network. In contrast, the COL-BS-EC group established a stable and structurally ordered network through the synergistic cross-linking between BS and EC. Specifically, the polyhydroxy groups in BS formed strong hydrogen bonds with collagen molecules, while its trace natural components, such as low molecular weight polyphenols, inhibited excessive collagen aggregation, thereby forming better-connected microchannels to facilitate water molecule penetration [29]. Concurrently, EC enhanced the cross-linking stability through interactions between phenolic hydroxyl groups and the amino groups of collagen [29]. This synergistic cross-linking mechanism not only significantly enhanced the network rigidity but also preserved sufficient pathways for water permeation, finally improving the mechanical properties and swelling behavior. In comparison, the collagen-sucrose-epicatechin group (COL-SUC-EC), due to the absence of trace active components, exhibited a storage modulus similar to that of the COL-EC group and a significantly lower swelling ratio (853%) than that of the COL-BS-EC group. This may be attributed to the synergistic effect of noncovalent bonds within the collagen–polyphenol composite hydrogel, which concurrently optimized the mechanical stability of the hydrogel network and the integrity of the hydrophilic channels [30].

The synergistic performance exhibited by the COL-BS-EC group held significant implications for wound dressing applications. The high swelling ratio ensured rapid absorption of wound exudate while maintaining a suitable moist microenvironment. The excellent mechanical properties ensured sufficient structural integrity during wound movement and minimized mechanical stimulation to newly formed tissue.

### Antibacterial properties of COL-BS-EC hydrogel

Burn wounds are highly susceptible to infection by bacteria such as Escherichia coli (*E. coli*) and Staphylococcus aureus (*S. aureus*) due to the disruption of skin barrier function and vascular damage [31]. Such infections not only intensify inflammatory responses but also lead to tissue necrosis and delay wound healing. In this study, antibacterial assays targeting these two prevalent bacteria in burn wounds demonstrated that EC serves as the core component responsible for the antibacterial properties of the hydrogels. Meanwhile, trace natural polyphenols present in brown sugar (BS) also exhibit a certain antibacterial effect, further enhancing the overall antibacterial properties of the hydrogels. This conclusion is supported by the differences in antibacterial ratios observed among the experimental groups (Figure 1F and G).

Specifically, all three hydrogel groups exhibited low antibacterial ratios against *E. coli* (Figure 1F): COL-BS (3.15% ± 1.01%), COL-BS-EC (7.93% ± 1.26%) and COL-SUC-EC (6.90% ± 1.08%), with no significant differences observed among them. In contrast, a gradient trend in antibacterial ratios was noted against *S. aureus* (Figure 1F): the COL-BS-EC group exhibited the highest ratio (43.34% ± 4.29%), followed by the COL-SUC-EC group (35.69% ± 4.63%), whereas the COL-BS group exhibited the lowest ratio (7.73% ± 0.88%).

The experimental data confirmed that EC represents the key component responsible for the antibacterial properties of the hydrogels. These hydrogels exhibited notable species-selective antibacterial properties; they showed generally low antibacterial ratios against *E. coli* (<10%) but distinctly stronger antibacterial effects against *S. aureus*. This selective antibacterial spectrum aligns closely with the intrinsic antibacterial profile of EC [32]. Notably, the COL-BS-EC group achieved a higher antibacterial ratio against *S. aureus* (43.34%) compared with the COL-SUC-EC group (35.69%). This improved activity is attributable to the additional antibacterial properties conferred by trace natural polyphenols (e.g. chlorogenic acid) present in BS, which are absent in pure sucrose (SUC) [33]. Consequently, the antibacterial properties demonstrated by the COL-BS-EC hydrogel present substantial potential for lowering the risk of burn wound infection and subsequent systemic complications.

The plate count method mainly evaluated the inhibition against planktonic bacteria, while the anti-biofilm efficacy was not directly tested. EC and the polyphenolic components in BS have been reported to significantly inhibit *S. aureus* biofilm formation. As a natural plant polyphenol, EC has been shown to significantly inhibit the formation of *S. aureus* biofilms and reduce the stability of mature biofilms by interfering with bacterial adhesion, suppressing the synthesis of extracellular polymeric substances (EPS) and downregulating the expression of quorum sensing (QS)-related genes [34]. Brown sugar, which is rich in polyphenols and flavonoids, can inhibit the initial adhesion of bacteria, disrupt the structure of biofilms and increase bacterial membrane permeability, thereby exerting an inhibitory effect on the formation of wound-associated pathogenic bacterial biofilms [35]. Furthermore, the antimicrobial evaluation will be further verified in future research.

### Antioxidant properties of COL-BS-EC hydrogel

Burn wounds continuously generate substantial quantities of reactive oxygen species (ROS), leading to severe oxidative stress that damages cellular structures and functions while impeding normal skin repair processes [36, 37]. EC, a natural antioxidant with excellent biocompatibility, has been demonstrated to be a safe and efficacious ROS scavenger [38].

**Figure 2:**
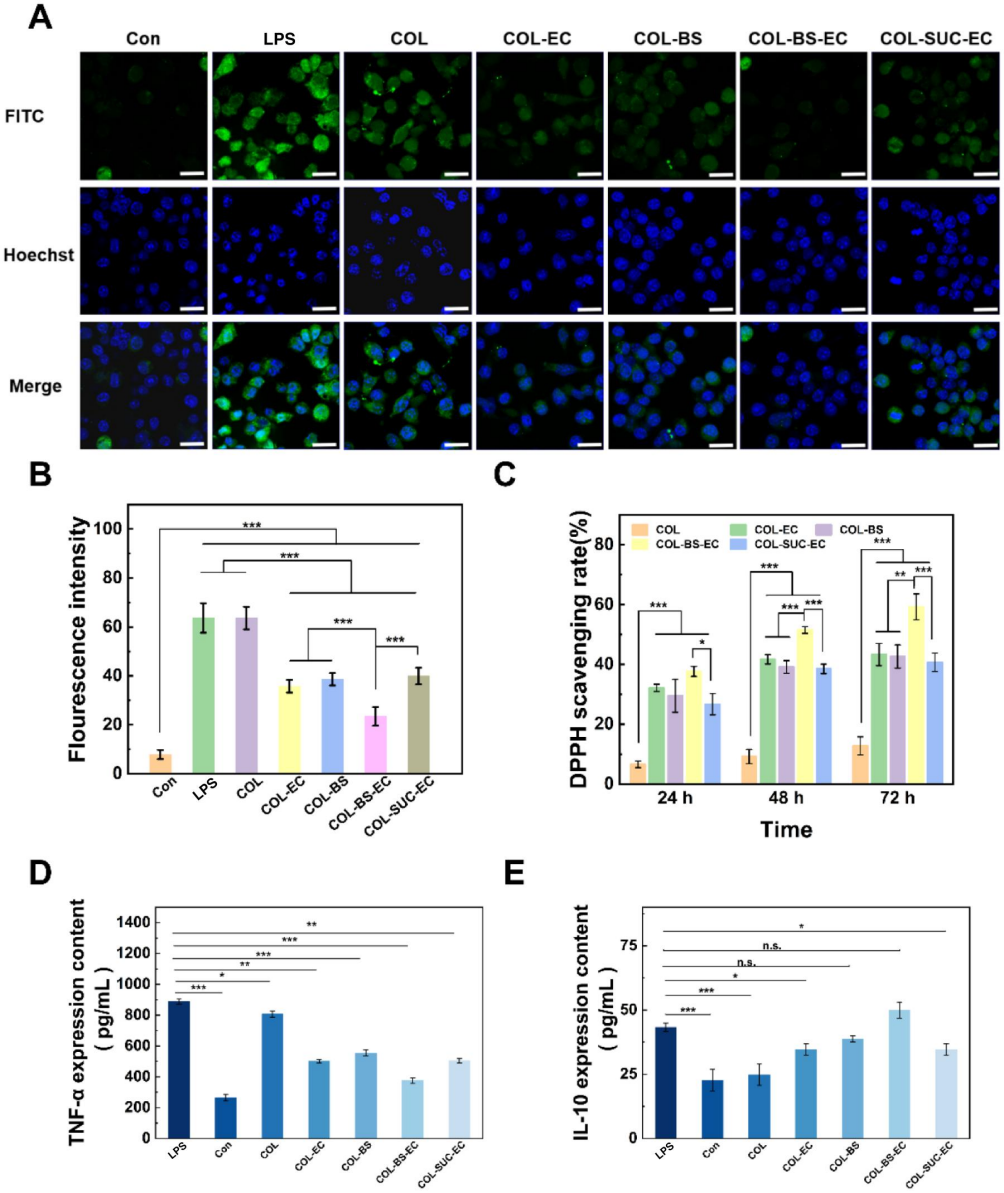
(**A**) CLSM images of RAW264.7 cells and (**B**) the semiquantitative fluorescence analysis of these images (bar = 20 μm). (**C**) DPPH radical scavenging ratio of hydrogel. (**D**) TNF-α and (**E**) IL-10 expression. *P < 0.05, **P < 0.01, ***P < 0.001, *n.s*.: no significance.

Experimental results (Figure 2C) demonstrated that DPPH radical scavenging rates in all hydrogel groups increased in a time-dependent manner. After 72 h, the COL-BS-EC group achieved the highest scavenging efficiency of 59.27% ± 4.35%, which was significantly higher than that of the COL group (12.77% ± 3.04%), COL-EC group (43.29% ± 3.72%), COL-BS group (42.64% ± 3.90%) and COL-SUC-EC group (40.71% ± 3.08%). Specifically, the DPPH scavenging rates of the COL-BS-EC group reached 37.64%, 51.52% and 59.27% at 24 h, 48 h and 72 h, respectively, while those of the COL-BS group were 29.55%, 39.16% and 42.64%, respectively. The antioxidant activity of both groups was significantly enhanced over time, with a gradual release profile, which demonstrates that the active components slowly diffused through the hydrogel network and exerted sustained effects.

Intracellular ROS scavenging experiments, including fluorescence imaging (Figure 2A) and semi-quantitative analysis (Figure 2B), further confirmed the superior antioxidant capacity of the COL-BS-EC treatment group. Specifically, ROS levels in the LPS-stimulated positive control group (63.66 ± 6.04) were markedly increased compared with the unstimulated negative control (7.84 ± 1.89). The COL-BS-EC treatment group (23.42 ± 3.79) exhibited the strongest intracellular ROS scavenging activity, significantly outperforming all other experimental groups.

This superior antioxidant performance can be attributed to two main factors. The primary factor is the potent antioxidant nature of EC as a natural polyphenol, which enables direct and efficient quenching of DPPH radicals through hydrogen atom or electron donation. The secondary factor is the additional free radical scavenging activity contributed by trace bioactive constituents (e.g. polyphenols and flavonoids) present in BS [39]. The stable incorporation and sustained release of EC and the bioactive components of BS within the collagen matrix not only facilitate direct free radical neutralization but also assist in regulating intracellular redox homeostasis. For burn wound applications, this synergistic antioxidant effect effectively mitigates excessive ROS-induced cellular apoptosis and extracellular matrix degradation, thereby creating a favorable microenvironment for tissue regeneration [5]. Moreover, the significantly enhanced antioxidant performance of the COL-BS-EC group compared with the COL-SUC-EC group substantiates the functional advantages of the bioactive components retained in primary brown sugar.

### Anti-inflammatory properties of COL-BS-EC hydrogel

Burn injuries typically elicit persistent inflammatory responses, which constitute a principal impediment to tissue regenerative healing [40]. ELISA analysis of TNF-α and IL-10 expression levels in RAW264.7 cells (Figure 2D and E) revealed that the COL-BS-EC group exhibited significantly lower expression of the pro-inflammatory cytokine TNF-α (375.28 ± 18.74 pg/mL) compared with the COL (807.10 ± 20.73 pg/mL), COL-EC (501.11 ± 10.84 pg/mL), COL-BS (554.88 ± 18.94 pg/mL) and COL-SUC-EC (504.77 ± 15.80 pg/mL) groups (Figure 2D). Meanwhile, the expression of the anti-inflammatory cytokine IL-10 was highest in the COL-BS-EC group (49.95 ± 3.16 pg/mL), significantly surpassing all other experimental groups (Figure 2E).

These results indicated that the COL-BS-EC hydrogel effectively remodeled the inflammatory microenvironment of burn wounds by downregulating pro-inflammatory cytokines and upregulating anti-inflammatory mediators. Inflammatory responses represent the major obstacle to burn wound regenerative healing, wherein TNF-α drives pro-inflammatory cascades while IL-10 promotes inflammatory resolution [41]. EC inhibits the activation of the NF-κB signaling pathway and reduces TNF-α secretion [42]. The bioactive constituents present in BS may further enhance this anti-inflammatory effect through synergistic suppression of pro-inflammatory cytokine release, while the collagen matrix provides a stable platform for the sustained release of these bioactive components, ensuring their prolonged therapeutic efficacy [43]. The superior anti-inflammatory performance demonstrated by the COL-BS-EC group confirms the synergistic interaction between EC and BS, which effectively balances inflammatory responses and thereby eliminates a critical pathological barrier to the regenerative repair of burn tissue.

### Cell migration-promoting capability of COL-BS-EC hydrogel

**Figure 3:**
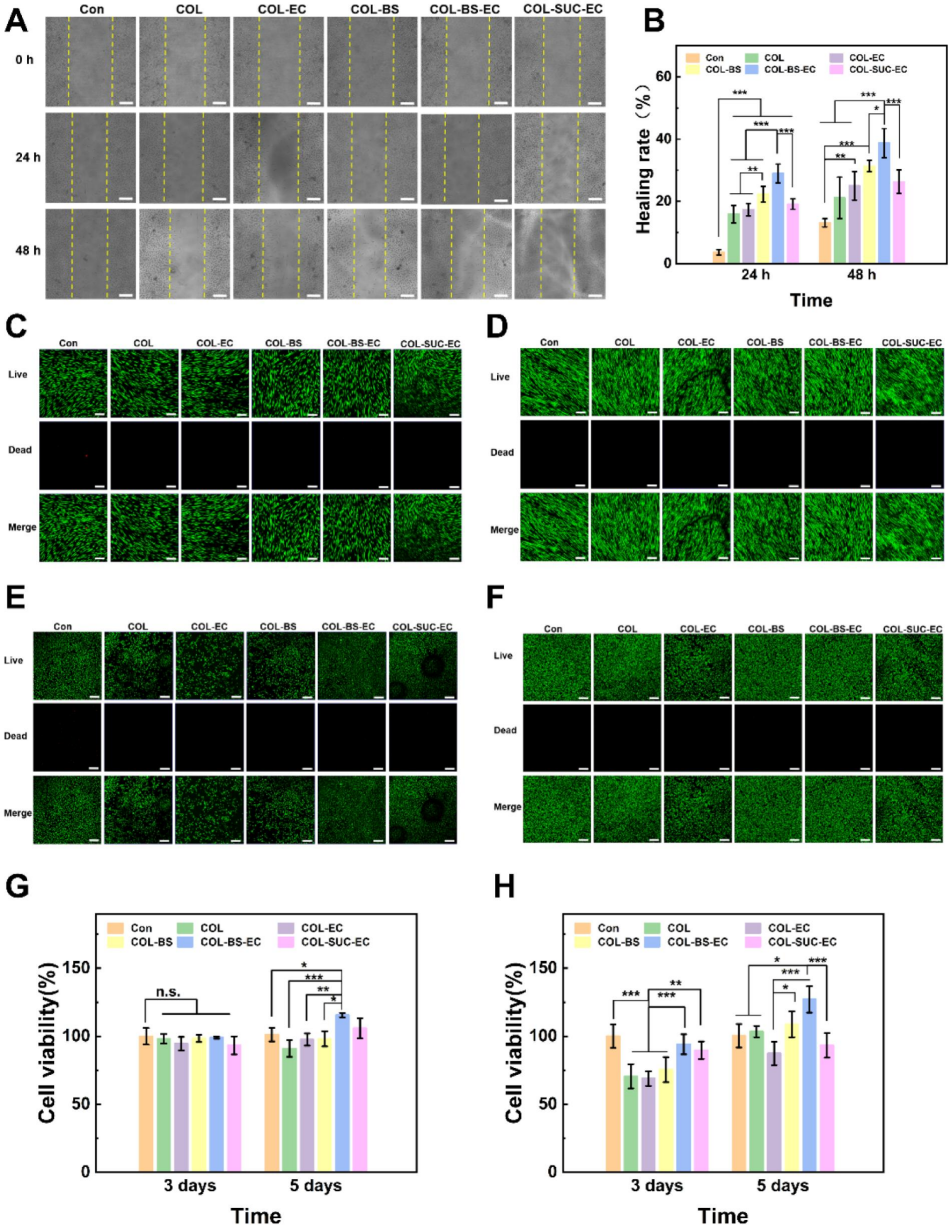
(**A**) Wound healing images and (**B**) healing rates of L929 cells (bar = 200 μm). The live/dead staining CLSM images of HDF cells cultured on the surface of hydrogel on Day 3 (**C**) and Day 5 (**D**) (bar = 200 μm). The live/dead staining CLSM images of L929 cells cultured on the surface of hydrogel on Day 3 (**E**) and Day 5 (**F**) (bar = 200 μm). Cell viability of HDF (**G**) and L929 (**H**) on Days 3 and 5. *P < 0.05, **P < 0.01, ***P < 0.001, *n.s*.: no significance.

Cell migration is a critical early phase of burn wound healing. Rapid fibroblast migration facilitates wound contraction and reduces the risk of infection [44]. In this study, the pro-migratory capacity of the hydrogels was evaluated using L929 fibroblast scratch-wound assays. The results demonstrated that the COL-BS-EC group significantly promoted cell migration (Figure 3A). Specifically, after 24 h (Figure 3B), this group exhibited the highest scratch healing rate (28.95% ± 3.01%), which was significantly higher than those of the blank control (3.63% ± 0.88%), COL (15.85% ± 2.82%), COL-EC (17.29% ± 2.03%), COL-BS (22.27% ± 2.58%) and COL-SUC-EC (19.10% ± 1.69%) groups. After 48 h (Figure 3B), the COL-BS-EC group maintained the highest scratch healing rate (38.75% ± 4.66%), which remained significantly higher than those of all other experimental groups.

The enhanced migratory activity observed with the COL-BS-EC hydrogel can be attributed to synergistic interactions among collagen, EC and BS. Specifically, collagen, as the principal structural protein of the extracellular matrix, provides an essential scaffold for cell adhesion and migration, thereby guiding directional cell movement [45]. The natural polyphenol EC directly enhances cell motility and promotes filopodial extension [46]. Additionally, non-sucrose components in BS, such as polyphenols and trace elements, optimize the microenvironment for cell migration [20, 43]. The improved effect compared with the COL-SUC-EC group stems primarily from the bioactive non-sucrose components present in BS. Pure sucrose lacks these functional components and therefore cannot establish effective synergism with EC and collagen to promote cell migration. Collectively, the cell migration-promoting activity demonstrated by the COL-BS-EC group creates favorable conditions for accelerating early-stage healing of burn wounds.

### Cell proliferation-promoting capacity of COL-BS-EC hydrogel

Cell proliferation is essential for granulation tissue formation and angiogenesis during burn wound repair [47]. In this study, cells were directly seeded onto hydrogel surfaces to better simulate the actual contact between clinical wound dressings and wounds. Live/dead staining and CCK-8 assay results demonstrated that the COL-BS-EC hydrogel significantly promoted the proliferation of both L929 cells and HDF cells, with the most significant effect observed on Day 5, confirming its capacity to support long-term cell growth requirements.

For HDF cells seeded on the hydrogel surfaces, no significant differences in proliferation rates were observed among the experimental groups after 3 days of culture (Figure 3C). However, on Day 5 (Figure 3D), the COL-BS-EC group exhibited the highest proliferation rate (Figure 3G; 115.54% ± 1.59%), which was significantly higher than those of the blank control (100.39% ± 8.62%), COL (90.91% ± 6.12%), COL-EC (97.60% ± 4.46%), COL-BS (98.12% ± 5.61%) and COL-SUC-EC (105.86% ± 7.45%) groups. For L929 cells, the proliferation rate of the COL-BS-EC (94.09% ± 7.34%) was already significantly higher than those of the COL (70.58% ± 8.87%) and COL-EC (68.87% ± 5.31%) groups on Day 3 (Figure 3E). On Day 5 (Figure 3F), the COL-BS-EC group maintained the highest proliferation rate (127.06% ± 9.70%; Figure 3H), significantly higher than those of the blank control (100.39% ± 8.62%), COL (103.43% ± 4.13%), COL-EC (87.41% ± 8.59%), COL-BS (108.80% ± 9.52%) and COL-SUC-EC (93.25% ± 9.05%) groups.

The pro-proliferative effects exhibited by the COL-BS-EC hydrogel may originate from a synergistic mechanism. Firstly, collagen not only provides a stable growth scaffold for cells, but its degradation products can also act as nutritional substrates to promote cell proliferation [48]. Secondly, EC can activate the ERK/MAPK signaling pathway and upregulate cyclin expression, thereby promoting cell cycle progression and stimulating the proliferation of both HDF and L929 cells [49]. Lastly, the non-sucrose components in BS exert a synergistic effect; for instance, minerals such as zinc act as essential cofactors for DNA polymerase and other proliferation-related enzymes [50], while polyphenolic constituents can promote cell proliferation by inhibiting apoptotic pathways [51].

The temporal differences in proliferation between L929 and HDF cells reflect the sustained-release property of the COL-BS-EC hydrogel. The collagen matrix enables continuous and gradual release of bioactive components from EC and BS, which is an important property for burn wound repair. The comparative results compared with the COL-SUC-EC group further confirmed the critical role of non-sucrose components in BS in promoting burn wound healing.

### *In vivo* evaluation for wound healing

Based on the theory of moist wound healing [52], the COL-BS-EC hydrogel, as a hydrated dressing enriched with bioactive macromolecules, facilitates the regenerative healing of burn wounds. This study systematically evaluated the therapeutic efficacy of the COL-BS-EC hydrogel in burn wound repair by using a deep second-degree burn model established in Kunming mice and rats. Two animal models were used to reduce species‑specific differences and improve the reliability of results. The experimental results demonstrated that the COL-BS-EC hydrogel significantly promoted burn wound healing.

**Figure 4:**
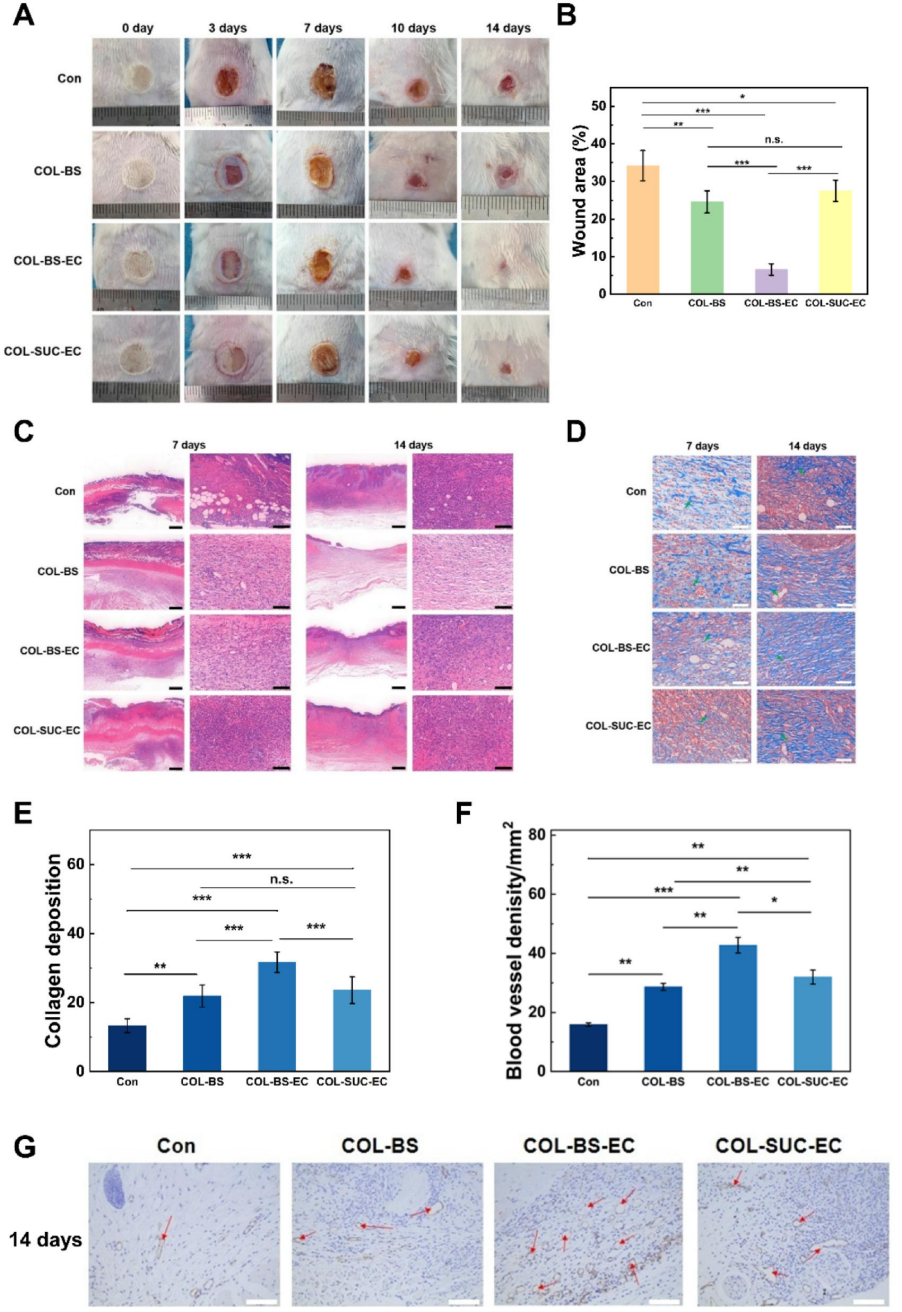
(**A**) Photographs of burn wounds at predetermined times of different treatment groups in mice model. (**B**) The percentage of wound area on Day 14. (**C**) H&E staining (bar = 400 μm, 100 μm) and (**D**) Masson staining (bar = 50 μm) of the wound sites on Day 7 and Day 14. Green arrows: collagen fiber. (**E**) Collagen deposition was measured on Day 14. (**F**) Number of newly formed blood vessels from the immunohistochemical images on Day 14. (**G**) CD31 immunohistochemically staining on Day 14 (bar = 100 μm). Red arrows: blood vessels. (*n *= 3) *P < 0.05, **P < 0.01, ***P < 0.001, *n.s*.: no significance.

Wound images (Figure 4A) and quantitative area analysis (Figure 4B) in the mice model revealed that after 7 days of treatment, the wound areas in all hydrogel-treated groups were significantly reduced compared with the control group. After 14 days of treatment, the newly formed tissue in the COL-BS-EC group nearly completely covered the original wound area. During the repair period, the COL-BS-EC hydrogel group demonstrated significantly higher healing rates compared with all other groups, with the regenerated skin tissue exhibiting morphological characteristics highly similar to those of normal skin. On Day 14, the residual wound area in the COL-BS-EC hydrogel group decreased to 6.61% ± 1.52% (Figure 4B), which was significantly lower than that in the control group (34.21% ± 4.01%), COL-BS group (24.56% ± 2.95%) and COL-SUC-EC group (27.55% ± 2.83%).

Histological analysis (Figure 4C) was further performed to evaluate skin tissue regeneration in burn wounds after hydrogel treatment. Evaluation of skin tissue repair revealed that more obvious tissue hyperplasia was observed on Day 14 compared with Day 7 in the control, COL-BS, COL-BS-EC and COL-SUC-EC groups, indicating improvement in tissue repair over time. On Day 7, the COL-BS, COL-BS-EC and COL-SUC-EC groups all exhibited increased inflammatory cell infiltration and fibrous tissue hyperplasia compared with the control group, signifying the entry into the tissue repair process. On Day 14, all treatment groups demonstrated significantly reduced inflammatory cell infiltration compared with the control group. However, the COL-SUC-EC group showed limited and disorganized fibrous tissue hyperplasia, while the COL-BS group showed moderate fibrous tissue formation with moderate organization. The COL-BS-EC group showed neatly arranged collagen fibers with an intact epidermal layer, indicating optimal tissue repair quality.

Collagen constitutes a critical component of extracellular matrix remodeling during the later phases of wound healing, and its substantial deposition effectively promotes fibroblast proliferation and extracellular matrix synthesis [53]. Masson staining revealed initial collagen deposition in all treatment groups on Day 7, with markedly increased areas of newly formed collagen fibers observed on Day 14. Compared with the control group, the COL-BS-EC hydrogel group exhibited denser and more structurally organized collagen deposition (Figure 4E). Quantitative analysis of collagen deposition demonstrated that the collagen content in the COL-BS-EC group (31.70% ± 2.95%) was significantly higher than those in the control group (13.30% ± 2.00%), COL-BS group (21.90% ± 3.20%) and COL-SUC-EC group (23.61% ± 3.87%). These results collectively indicate the superior wound healing efficacy of the COL-BS-EC hydrogel. The present study evaluated total collagen content and arrangement by Masson staining; the assessment of the Type I/III collagen ratio was not performed. However, the complete epidermal structure, orderly collagen arrangement and high neovascular density in COL-BS-EC group indirectly reflect improved regeneration quality. Epicatechin and protocatechuic acid in brown sugar have been reported to regulate collagen metabolism and improve the Type I/III ratio [54–56]. In future work, picrosirius red staining, immunofluorescence double labeling and qPCR will be used to quantify Type I and III collagen and their ratio for more comprehensive regeneration evaluation.

Furthermore, wound healing depends on neovascularization for delivering essential nutrients and clearing metabolic waste [57], and angiogenesis is a critical indicator for evaluating healing progression [58]. CD31 immunohistochemical analysis (Figure 4G) revealed neovascular formation in all groups on Day 7, with the control group showing the least neovascularization and the COL-BS-EC hydrogel group showing the highest vascular density. On Day 14, the COL-BS-EC group maintained a significantly higher number of newly formed blood vessels compared with all other groups. CD31 immunofluorescence quantification analysis (Figure 4F) demonstrated that the number of newly formed vessels in the COL-BS-EC group (42.75 ± 2.64 vessels/mm^2^) was significantly higher than those in the control group (15.91 ± 0.56 vessels/mm^2^), COL-BS group (28.66 ± 1.18 vessels/mm^2^) and COL-SUC-EC group (32.00 ± 2.37 vessels/mm^2^), confirming its superior pro-angiogenic activity. These findings collectively demonstrate that the COL-BS-EC hydrogel effectively promotes the regenerative healing of burn wounds.

In the animal experiment, hydrogel dressings were replaced every 2 days. Throughout the 14-day treatment period, the COL-BS-EC group consistently exhibited the optimal healing efficacy. Particularly during the late treatment stage, the wound area continued to decrease, with continuous improvements in collagen deposition and neovascularization. This trend suggests that the hydrogel maintained sufficient structural integrity and bioactivity during the every 2-day dressing replacement interval. Therefore, the release of active components from the hydrogel encompassed this time window at least, with no evidence of burst release followed by rapid loss of efficacy.

**Figure 5:**
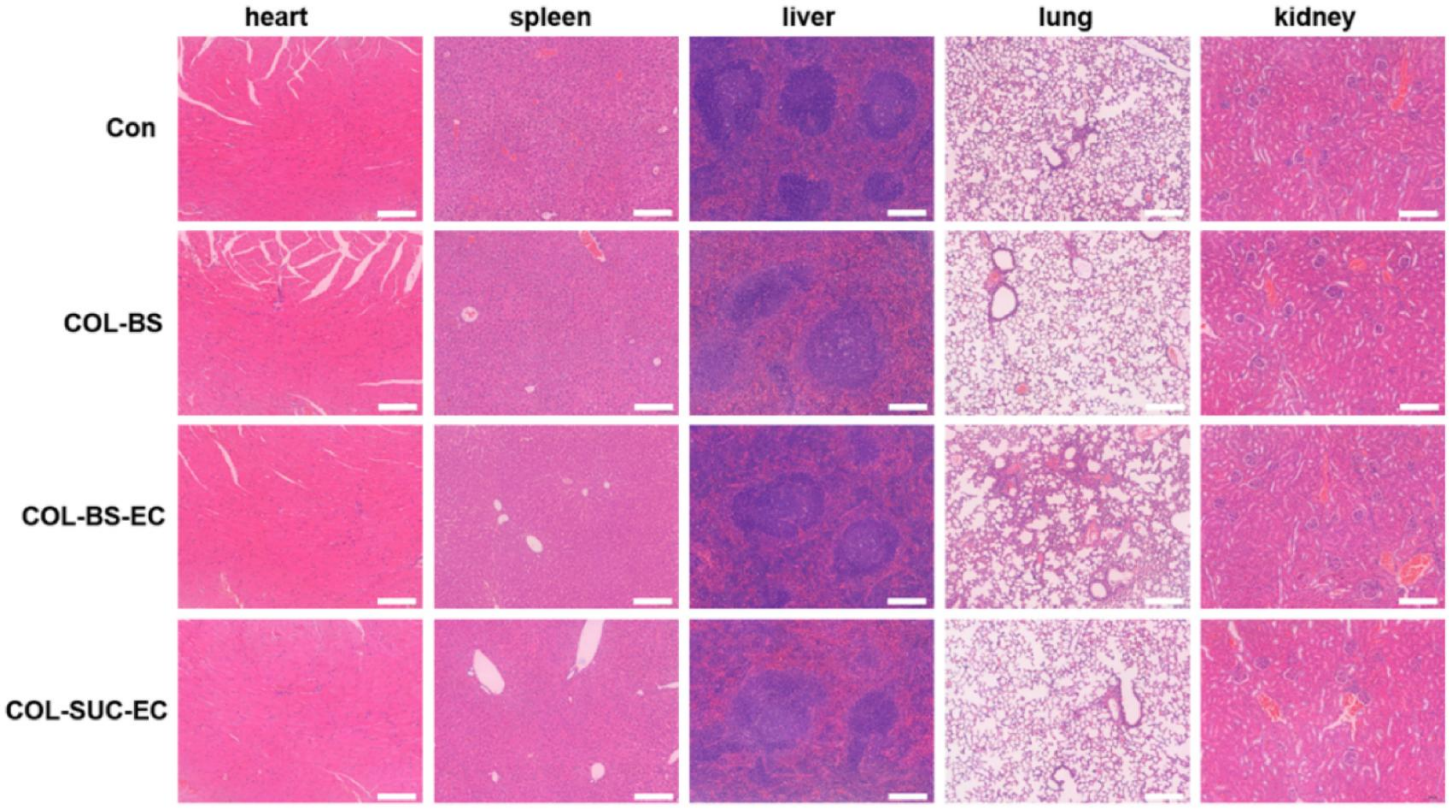
*In vivo* biocompatibility examination of hydrogels. H&E staining (bar = 200 μm) on Day 14 of the main organs (heart, liver, spleen, lung and kidney).

Systematic evaluation of *in vivo* biosafety is essential for the clinical translation of novel medical wound dressings [59]. In this study, the preparation of the composite hydrogels was performed entirely under aseptic conditions with filtered brown sugar solution to ensure the inherent sterility of the final hydrogel dressings. Comprehensive histological analysis was performed on major organs (heart, liver, spleen, lung and kidney) harvested from mice following 14 days of COL-BS-EC hydrogel treatment. The results (Figure 5) demonstrated that all major organs retained normal histoarchitecture, with no significant pathological alterations such as inflammatory infiltration, necrosis or fibrosis, thereby confirming excellent biocompatibility of the COL-BS-EC hydrogel. During the entire treatment period, no obvious erythema or edema was observed at the wound sites or in the surrounding skin, indicating that the hydrogel dressings possess no significant sensitization effects.

**Figure 6:**
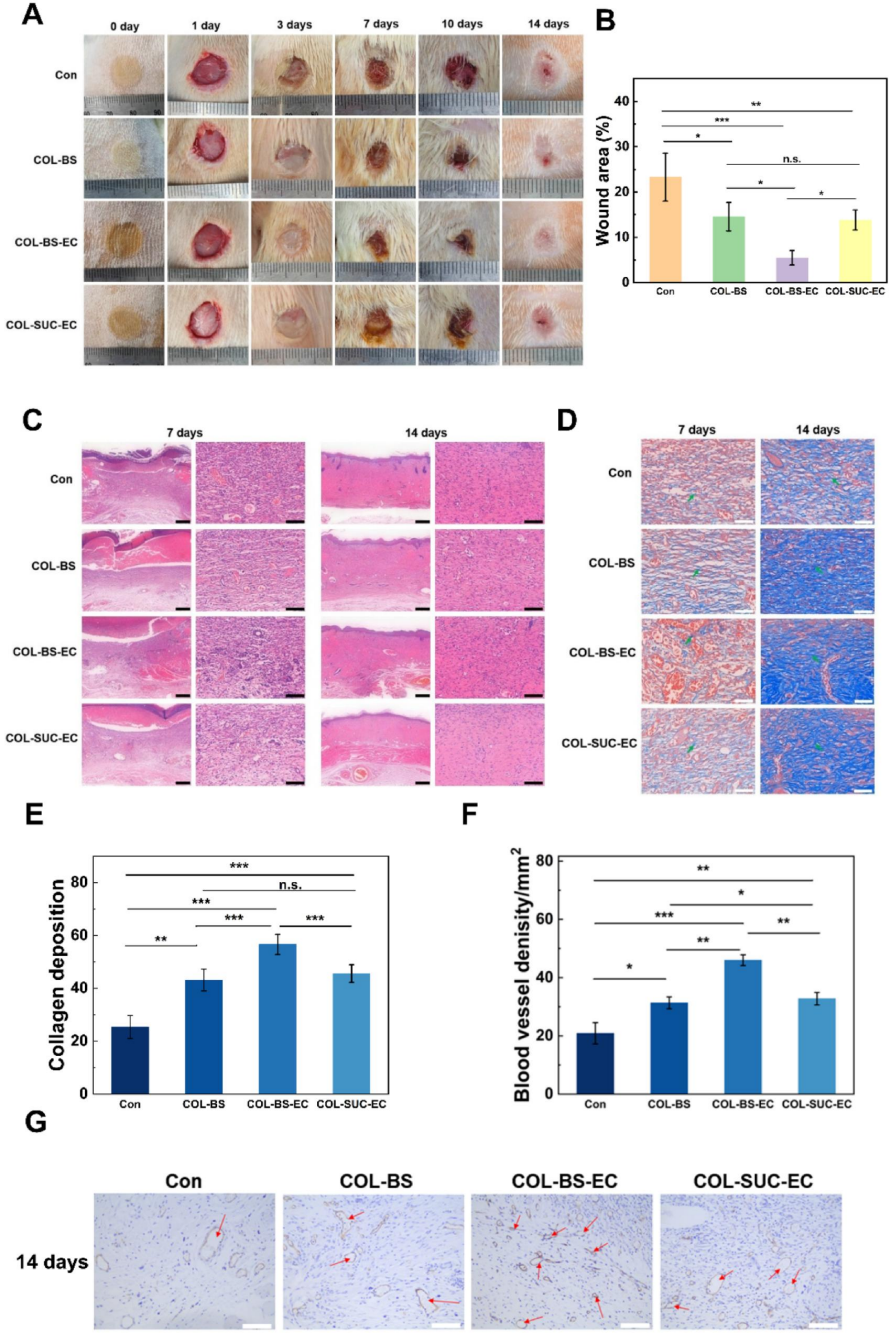
(**A**) Photographs of burn wounds at predetermined times of different treatment groups in rat model. (**B**) The percentage of wound area on Day 14. (**C**) H&E staining (bar = 400 μm, 100 μm) and (**D**) Masson staining (bar = 50 μm) of the wound sites on Day 7 and Day 14. Green arrows: collagen fiber. (**E**) Collagen deposition was measured on Day 14. (**F**) Number of newly formed blood vessels from the immunohistochemical images on Day 14. (**G**) CD31 immunohistochemically staining on Day 14 (bar = 100 μm). Red arrows: blood vessels. (*n = *3) *P < 0.05, **P < 0.01, ***P < 0.001, n.s.: no significance.

To further validate the therapeutic efficacy and eliminate potential interference from individual variations, a replicate experiment was conducted using a rat burn wound model. The results (Figure 6A–G) exhibited similar healing trends to those of the mouse model, the COL-BS-EC hydrogel group exhibiting significantly accelerated wound healing compared with the other groups. On Day 14, the residual wound area in this group decreased to only 5.47% of the original wound area (Figure 6A), and histological analysis (Figure 6B–G) further confirmed its capacity to effectively promote the regenerative healing of burn wounds.

Two animal models (mouse and rat) were used to enhance result reliability. Mice have smaller body sizes and weaker spontaneous skin repair capacity, which enables a more sensitive assessment of the hydrogel’s wound-healing-promoting efficacy. In the mouse model, only one wound was created per animal and treated with the same hydrogel throughout the study, allowing for a more accurate evaluation of the material’s bio-safety. In contrast, for the rat model, four wounds were created on the dorsum of each rat and treated with normal saline or the hydrogels from different groups. This within-subject design effectively eliminates inter-individual variability. Meanwhile, the skin thickness, healing rate and immune response are different among different species. The inclusion of two distinct animal models mitigates potential confounding effects arising from species-specific differences and inter-individual physiological variations, thereby validating the stability and reproducibility of the wound-healing-promoting effects of the COL-BS-EC hydrogel. These findings provide a valuable reference for subsequent large-animal studies and offer more robust preclinical evidence for translational research.

In conclusion, these findings not only substantiate the excellent *in vivo* safety profile of the COL-BS-EC hydrogel but also confirm its considerable potential as a safe clinical wound dressing through its consistent therapeutic efficacy across distinct animal models.

In this study, the synergistic contributions of non-sucrose components were primarily established through comparative evaluation of control groups (COL, COL-EC, COL-BS, COL-BS-EC and COL-SUC-EC) in terms of antibacterial, antioxidant, anti-inflammatory, pro-migratory and pro-proliferative activities, as well as wound healing results in animal models. Epicatechin has been reported to inhibit NF-κB pathway activation and suppress the phosphorylation of ERK, JNK and p38 in the MAPK cascade, thus exerting anti-inflammatory and pro-migratory effects [32, 46]. The collagen matrix regulates cell behaviors via integrin-mediated PI3K/Akt, FAK and TGF-β/Smad signaling pathways to promote fibroblast proliferation and ECM remodeling [53, 60]. Non-sucrose constituents such as protocatechuic acid in brown sugar can inhibit p38 MAPK and NF-κB pathways, while zinc and magnesium serve as cofactors involved in MAPK and PI3K/Akt signaling [43, 50]. The significantly better performance of COL-BS-EC than COL-SUC-EC in reducing TNF-α and elevating IL-10, as well as the highest neovascular density, suggests the synergistic effect of brown sugar non-sucrose components and EC through NF-κB, MAPK and related pathways. In-depth molecular mechanistic studies including qPCR, Western blot and immunofluorescence targeting p-NF-κB/p65, p-IκBα, p-ERK, p-p38, p-JNK and p-STAT3 will be conducted in future work to provide direct evidence.

In this work, the active collagen-based hydrogel fabricated according to this new design concept was investigated by establishing comprehensive experimental controls. Physicochemical characterization (including component analysis, SEM imaging, mechanical performance, swelling behavior and adhesive properties) was evaluated to demonstrate the operational convenience required for clinical application. *In vitro* cellular experiments (including DPPH free radical scavenging, intracellular ROS elimination, antibacterial and anti-inflammatory assays) were conducted to verify the essential functionalities expected of a wound dressing material, namely antioxidant, antibacterial and anti-inflammatory capacities. *In vivo* dual animal models (mouse and rat) were established to directly confirm the favorable wound repair efficacy and validate the feasibility of this biomaterial design concept.

## Conclusions

Inspired by the concept of synergistic treatment in traditional Chinese medicine, BS, as the primary extract from sugarcane, was introduced into the collagen solution along with EC to obtain a collagen-based composite hydrogel through the gelation behavior derived from the self-assembly of collagen macromolecules. This hydrogel aimed at being designed as a dressing material with improved functions for the therapy of severe skin burns. Since BS comprises diverse functional components, the utilization of brown sugar in surgical materials embodied the concept of formula-based therapy, which was confirmed not only by cytological and biological examinations but also by *in vivo* experiments employing comprehensive control groups of COL, COL-EC, COL-BS and COL-SUC-EC, thus highlighting the synergistically promoting healing effects contributed from the sucrose and non-sucrose components within BS for burn wounds.

Results of characterizations showed that the composite hydrogel of COL-BS-EC possesses a three-dimensional porous structure, appropriate swelling behavior and improved mechanical strength, providing an ideal structural foundation for nutrient exchange and maintaining a moist wound-healing microenvironment. Biologically, this hydrogel exhibited potent antioxidant activity of efficiently scavenging DPPH free radicals and inhibiting intracellular ROS accumulation. Its significant antibacterial activity against *S. aureus* was also identified. Furthermore, the phenomenon of precise modulation of theinflammatory microenvironment by downregulating the pro-inflammatory factor TNF-α and upregulating the anti-inflammatory factor IL-10 also occurred for the COL-BS-EC group. Burn models on mouse and rat revealed that the hydrogel dressing of COL-BS-EC exhibited outstanding effects of acceleration for wound closure, promotion for organized collagen deposition and stimulation for functional angiogenesis in comparison to the group of COL-SUC-EC, demonstrably elucidating the mechanism of synergistic therapy dominantly given from BS.

This study is a proof-of-concept investigation aiming at discovering and preliminarily validating a multicomponent strategy via which bioactive collagen hydrogel was loaded with natural formulations, exemplified by brown sugar containing diverse constituents, to markedly improve burn wounds with regenerative healing originating from the synergistic effects. This approach validates the feasibility of this novel biomaterial design concept and provides a new design perspective for functional dressing materials enabling wounds with regenerative healing in shorter cycles.

## References

[1] Wang Y , Vizely K, Li CY, Shen K, Shakeri A, Khosravi R, Smith JR, Alteza EAII, Zhao Y, Radisic M. Biomaterials for immunomodulation in wound healing. Regen Biomater 2024;11:rbae032. 38779347 10.1093/rb/rbae032 PMC11110865

[2] Xiao M , Li L, Hu Q, Ma L, Liu L, Chu W, Zhang H. Rapamycin reduces burn wound progression by enhancing autophagy in deep second-degree burn in rats. Wound Repair Regen 2013;21:852–9. 23980869 10.1111/wrr.12090

[3] Gross-Amat O , Guillen M, Salmon D, Nataf S, Auxenfans C. Characterization of a topically testable model of burn injury on human skin explants. Int J Mol Sci 2020;21:6956. 32971882 10.3390/ijms21186956 PMC7554828

[4] Gamelli RL , Liu H, He LK, Hofmann CA. Augmentations of glucose uptake and glucose transporter-1 in macrophages following thermal injury and sepsis in mice. J Leukoc Biol 1996;59:639–47. 8656048 10.1002/jlb.59.5.639

[5] Parihar A , Parihar MS, Milner S, Bhat S. Oxidative stress and anti-oxidative mobilization in burn injury. Burns 2008;34:6–17. 17905515 10.1016/j.burns.2007.04.009

[6] Chakrabarti S , Mazumder B, Rajkonwar J, Pathak MP, Patowary P, Chattopadhyay P. bFGF and collagen matrix hydrogel attenuates burn wound inflammation through activation of ERK and TRK pathway. Sci Rep 2021;11:3357. 33558597 10.1038/s41598-021-82888-9 PMC7870886

[7] Singh O , Gupta SS, Soni M, Moses S, Shukla S, Mathur RK. Collagen dressing versus conventional dressings in burn and chronic wounds: a retrospective study. J Cutan Aesthet Surg 2011;4:12–6. 21572675 10.4103/0974-2077.79180 PMC3081477

[8] Knipe JM , Peppas NA. Multi-responsive hydrogels for drug delivery and tissue engineering applications. Regen Biomater 2014;1:57–65. 26816625 10.1093/rb/rbu006 PMC4669007

[9] Chen J , Cai Z, Wei Q, Wang D, Wu J, Tan Y, Lu J, Ai H. Proanthocyanidin-crosslinked collagen/konjac glucomannan hydrogel with improved mechanical properties and MRI trackable biodegradation for potential tissue engineering scaffolds. J Mater Chem B 2020;8:316–31. 31819938 10.1039/c9tb02053e

[10] Gu H , Li H, Wei L, Lu J, Wei Q. Collagen-based injectable and self-healing hydrogel with multifunction for regenerative repairment of infected wounds. Regen Biomater 2023;10:rbad018. 36974203 10.1093/rb/rbad018 PMC10039733

[11] Ng JY , Zhu X, Mukherjee D, Zhang C, Hong S, Kumar Y, Gokhale R, Ee PLR. Pristine gellan gum-collagen interpenetrating network hydrogels as mechanically enhanced anti-inflammatory biologic wound dressings for burn wound therapy. ACS Appl Bio Mater 2021;4:1470–82. 10.1021/acsabm.0c01363 35014496

[12] Rana MM , Rahman MS, Ullah MA, Siddika A, Hossain ML, Akhter MS, Hasan MZ, Asaduzzaman SM. Amnion and collagen-based blended hydrogel improves burn healing efficacy on a rat skin wound model in the presence of wound dressing biomembrane. Biomed Mater Eng 2020;31:1–17. 32144968 10.3233/BME-201076

[13] Li R , Xu Z, Jiang Q, Zheng Y, Chen Z, Chen X. Characterization and biological evaluation of a novel silver nanoparticle-loaded collagen-chitosan dressing. Regen Biomater 2020;7:371–80. 32793382 10.1093/rb/rbaa008 PMC7414998

[14] Emad NA , Zai I, Ahmad S, Pandit J, Khan MA, Sultana Y. Role of polyphenols, their nano-formulations, and biomaterials in diabetic wound healing. Endocr Metab Immune Disord Drug Targets 2024;24:626–41. 37817658 10.2174/0118715303242310230927104709

[15] Melguizo-Rodríguez L , de Luna-Bertos E, Ramos-Torrecillas J, Illescas-Montesa R, Costela-Ruiz VJ, García-Martínez O. Potential effects of phenolic compounds that can Be found in olive oil on wound healing. Foods 2021;10:1642. 34359512 10.3390/foods10071642 PMC8307686

[16] Monika P , Chandraprabha MN, Murthy KNC. Catechin, epicatechin, curcumin, garlic, pomegranate peel and neem extracts of Indian origin showed enhanced anti-inflammatory potential in human primary acute and chronic wound derived fibroblasts by decreasing TGF-β and TNF-α expression. BMC Complement Med Ther 2023;23:181. 37268940 10.1186/s12906-023-03993-y PMC10236757

[17] Li Y , Ma S, Zhang Y, Yao M, Zhu X, Guan F. (-)-epicatechin mitigates radiation-induced intestinal injury and promotes intestinal regeneration via suppressing oxidative stress. Free Radic Res 2019;53:851–64. 31234659 10.1080/10715762.2019.1635692

[18] Lohrey S , Chua M, Gros C, Faucet J, Lee JKW. Perceptions of heat-health impacts and the effects of knowledge and preventive actions by outdoor workers in Hanoi, Vietnam. Sci Total Environ 2021;794:148260. 34328123 10.1016/j.scitotenv.2021.148260

[19] Azlan A , Khoo HE, Sajak AAB, Aizan Abdul Kadir NA, Yusof BNM, Mahmood Z, Sultana S. Antioxidant activity, nutritional and physicochemical characteristics, and toxicity of minimally refined brown sugar and other sugars. Food Sci Nutr 2020;8:5048–62. 32994965 10.1002/fsn3.1803 PMC7500760

[20] Qiang Z , Shuai Y, Sicong Z, Yi L, Guanghui X, Qiang W, Bao W, Shiming Q, Bowei Z, Yinru H, Jian W. Analysis of nutritional components in brown sugar and its effects on anti-fatigue, alleviation of Qi deficiency and blood stasis, and antioxidant effects. bioRxiv 2025. 10.1101/2025.09.15.676350

[21] Gutsche N , Holtmannspötter M, Maß L, O’Donoghue M, Busch A, Lauri A, Schubert V, Zachgo S. Conserved redox-dependent DNA binding of ROXY glutaredoxins with TGA transcription factors. Plant Direct 2017;1:e00030. 31245678 10.1002/pld3.30 PMC6508501

[22] Sharma A , Schwartz SM, Méndez E. Hospital volume is associated with survival but not multimodality therapy in Medicare patients with advanced head and neck cancer. Cancer 2013;119:1845–52. 23456789 10.1002/cncr.27976 PMC6121709

[23] Shi L , Zhao W, Yang Z, Subbiah V, Suleria HAR. Extraction and characterization of phenolic compounds and their potential antioxidant activities. Environ Sci Pollut Res Int 2022;29:81112–29. 36201076 10.1007/s11356-022-23337-6 PMC9606084

[24] Criollo-Mendoza MS , Contreras-Angulo LA, Leyva-López N, Gutiérrez-Grijalva EP, Jiménez-Ortega LA, Heredia JB. Wound healing properties of natural products: mechanisms of action. Molecules 2023;28:598. 36677659 10.3390/molecules28020598 PMC9867334

[25] Ren P , Guan DW, Zhao R, Ma WX, Zhang ST. Establishment of skin scald model in mice. Fa Yi Xue Za Zhi 2012;28:92–4, 99. 22619801

[26] Feng W , Wang Z. Tailoring the swelling-shrinkable behavior of hydrogels for biomedical applications. Adv Sci (Weinh) 2023;10:e2303326. 37544909 10.1002/advs.202303326 PMC10558674

[27] Zhang C , Zhao H, Geng S, Li C, Liu J, Chen Y, Yi M, Liu Y, Guan F, Yao M. Adhesive, stretchable, and photothermal antibacterial hydrogel dressings for wound healing of infected skin burn at joints. Biomacromolecules 2024;25:7750–66. 39540762 10.1021/acs.biomac.4c01023

[28] Grant CA , Phillips MA, Thomson NH. Dynamic mechanical analysis of collagen fibrils at the nanoscale. J Mech Behav Biomed Mater 2012;5:165–70. 22100091 10.1016/j.jmbbm.2011.08.020

[29] Munir S , Yue W, Li J, Yu X, Ying T, Liu R, You J, Xiong S, Hu Y. Effects of phenolics on the physicochemical and structural properties of collagen hydrogel. Polymers (Basel) 2023;15:4647. 38139899 10.3390/polym15244647 PMC10747534

[30] Liu WC , Wang HY, Lee TH, Chung RJ. Gamma-poly glutamate/gelatin composite hydrogels crosslinked by proanthocyanidins for wound healing. Mater Sci Eng C Mater Biol Appl 2019;101:630–9. 31029356 10.1016/j.msec.2019.04.018

[31] Wang Y , Beekman J, Hew J, Jackson S, Issler-Fisher AC, Parungao R, Lajevardi SS, Li Z, Maitz PKM. Burn injury: challenges and advances in burn wound healing, infection, pain and scarring. Adv Drug Deliv Rev 2018;123:3–17. 28941987 10.1016/j.addr.2017.09.018

[32] Yang Y , Zhang T. Antimicrobial activities of tea polyphenol on phytopathogens: a review. Molecules 2019;24:816. 30823535 10.3390/molecules24040816 PMC6413138

[33] Juan L , You-feng L, HU X-L, Zhi P, QU J-N Study on extraction of chlorogenic acid and determination of antibacterial activity of its rare-earth complexes. Chem Ind For Prod 2007;27:85–8.

[34] Wang S , Chang Y, Xu J, Zhao C, Song Y, Liu M, Tian C. Comparative study of anti-inflammatory, antioxidant and antibacterial activities of epigallocatechin gallate, epigallocatechin, epicatechin gallate and epicatechin in vitro. Pharmacol Res - Mod Chin Med 2025;15:100599.

[35] Barrera C , Betoret N, Seguí L. Phenolic profile of cane sugar derivatives exhibiting antioxidant and antibacterial properties. Sugar Tech 2020;22:798–811.

[36] Chen R , Xu H, Li X, Dong J, Wang S, Hao J, Liang G. Role of oxidative stress in post-burn wound healing. Burns Trauma 2025;13:tkaf040. 40979899 10.1093/burnst/tkaf040 PMC12449078

[37] Zhao X , Dai W, Liu C, An M, Li S, Guo L, Fan Y, Zhang X. Gelatin/hyaluronic acid-based in situ forming hydrogel promotes wound regeneration by the synergy of ROS-scavenging and pro-healing activity. Regen Biomater 2025;12:rbaf052. 40703206 10.1093/rb/rbaf052 PMC12286701

[38] Prakash M , Basavaraj BV, Chidambara Murthy KN. Biological functions of epicatechin: plant cell to human cell health. J Funct Foods 2019;52:14–24.

[39] Zhu Z , Xie C, Li W, Hang F, Li K, Shi C, Doherty WOS. Nutritional and antioxidant properties of non-centrifugal cane sugar derived from membrane clarified juice. LWT 2020;131:109717.

[40] Yang J , Huang Y, Dai J, Shi X, Zheng Y. A sandwich structure composite wound dressing with firmly anchored silver nanoparticles for severe burn wound healing in a porcine model. Regen Biomater 2021;8:rbab037. 34350029 10.1093/rb/rbab037 PMC8329475

[41] Reuter S , Gupta SC, Chaturvedi MM, Aggarwal BB. Oxidative stress, inflammation, and cancer: how are they linked? Free Radic Biol Med 2010;49:1603–16. 20840865 10.1016/j.freeradbiomed.2010.09.006 PMC2990475

[42] Truong V-L , Jeong W-S. Antioxidant and anti-inflammatory roles of tea polyphenols in inflammatory bowel diseases. Food Sci Hum Wellness 2022;11:502–11.

[43] Ontawong A , Duangjai A, Vaddhanaphuti CS, Amornlerdpison D, Pengnet S, Kamkaew N. Chlorogenic acid rich in coffee pulp extract suppresses inflammatory status by inhibiting the p38, MAPK, and NF-κB pathways. Heliyon 2023;9:e13917. 36873494 10.1016/j.heliyon.2023.e13917 PMC9982044

[44] Zhang P , Zou B, Liou YC, Huang C. The pathogenesis and diagnosis of sepsis post burn injury. Burns Trauma 2021;9:tkaa047. 33654698 10.1093/burnst/tkaa047 PMC7901709

[45] Zhou H , Li W, Pan L, Zhu T, Zhou T, Xiao E, Wei Q. Human extracellular matrix (ECM)-like collagen and its bioactivity. Regen Biomater 2024;11:rbae008. 38545260 10.1093/rb/rbae008 PMC10965421

[46] Qu Z , Liu A, Li P, Liu C, Xiao W, Huang J, Liu Z, Zhang S. Advances in physiological functions and mechanisms of (-)-epicatechin. Crit Rev Food Sci Nutr 2021;61:211–33. 32090598 10.1080/10408398.2020.1723057

[47] Smith J , Rai V. Novel factors regulating proliferation, migration, and differentiation of fibroblasts, keratinocytes, and vascular smooth muscle cells during wound healing. Biomedicines 2024;12:1939. 39335453 10.3390/biomedicines12091939 PMC11429312

[48] Ohara H , Ichikawa S, Matsumoto H, Akiyama M, Fujimoto N, Kobayashi T, Tajima S. Collagen-derived dipeptide, proline-hydroxyproline, stimulates cell proliferation and hyaluronic acid synthesis in cultured human dermal fibroblasts. J Dermatol 2010;37:330–8. 20507402 10.1111/j.1346-8138.2010.00827.x

[49] Schroeter H , Bahia P, Spencer JP, Sheppard O, Rattray M, Cadenas E, Rice-Evans C, Williams RJ. (-)epicatechin stimulates ERK-dependent cyclic AMP response element activity and up-regulates GluR2 in cortical neurons. J Neurochem 2007;101:1596–606. 17298385 10.1111/j.1471-4159.2006.04434.x

[50] Holtzen SE , Navid E, Kainov JD, Palmer AE. Transient Zn^2+^ deficiency induces replication stress and compromises daughter cell proliferation. Proc Natl Acad Sci USA 2024;121:e2321216121. 38687796 10.1073/pnas.2321216121 PMC11087780

[51] Wang S , DeGroff VL, Clinton SK. Tomato and soy polyphenols reduce insulin-like growth factor-I–stimulated rat prostate cancer cell proliferation and apoptotic resistance in vitro via inhibition of intracellular signaling pathways involving tyrosine kinase. J Nutr 2003;133:2367–76. 12840208 10.1093/jn/133.7.2367

[52] Nuutila K , Eriksson E. Moist wound healing with commonly available dressings. Adv Wound Care (New Rochelle) 2021;10:685–98. 32870777 10.1089/wound.2020.1232 PMC8568799

[53] Mathew-Steiner SS , Roy S, Sen CK. Collagen in wound healing. Bioengineering (Basel) 2021;8:63. 34064689 10.3390/bioengineering8050063 PMC8151502

[54] Miranda Buendia E , González-Gómez GH, Maciel-Cerda A, González-Torres M. Epicatechin derivatives in tissue engineering: antioxidant, anti-inflammatory, regenerative use. Tissue Eng B Rev 2025;31:504–16. 10.1089/ten.teb.2024.0206 39656100

[55] Lee KE , Bharadwaj S, Yadava U, Kang SG. Computational and in vitro investigation of (-)-epicatechin and proanthocyanidin B2 as inhibitors of human matrix metalloproteinase 1. Biomolecules 2020;10:1379. 32998374 10.3390/biom10101379 PMC7650666

[56] Silva S , Michniak-Kohn B, Leonardi GR. An overview about oxidation in clinical practice of skin aging. An Bras Dermatol 2017;92:367–74. 29186250 10.1590/abd1806-4841.20175481 PMC5514578

[57] Shi Z , Yao C, Shui Y, Li S, Yan H. Research progress on the mechanism of angiogenesis in wound repair and regeneration. Front Physiol 2023;14:1284981. 38089479 10.3389/fphys.2023.1284981 PMC10711283

[58] Moreira HR , Marques AP. Vascularization in skin wound healing: where do we stand and where do we go? Curr Opin Biotechnol 2022;73:253–62. 34555561 10.1016/j.copbio.2021.08.019

[59] Rosa V , Silikas N, Yu B, Dubey N, Sriram G, Zinelis S, Lima AF, Bottino MC, Ferreira JN, Schmalz G, Watts DC. Guidance on the assessment of biocompatibility of biomaterials: fundamentals and testing considerations. Dent Mater 2024;40:1773–85. 39129079 10.1016/j.dental.2024.07.020

[60] Ullah S , Zainol I. Fabrication and applications of biofunctional collagen biomaterials in tissue engineering. Int J Biol Macromol 2025;298:139952. 39824416 10.1016/j.ijbiomac.2025.139952

